# Production of Innovative Essential Oil-Based Emulsion Coatings for Fungal Growth Control on Postharvest Fruits

**DOI:** 10.3390/foods11111602

**Published:** 2022-05-29

**Authors:** Mahsa Sadat Razavi, Abdollah Golmohammadi, Ali Nematollahzadeh, Alireza Ghanbari, Mahdi Davari, Daniele Carullo, Stefano Farris

**Affiliations:** 1 Department of Biosystems Engineering, University of Mohaghegh Ardabili, Daneshgah Street, Ardabil 56199-11367, Iran; mahsarazavi.68@gmail.com; 2 Department of Chemical Engineering, University of Mohaghegh Ardabili, Daneshgah Street, Ardabil 56199-11367, Iran; nematollahzadeha@uma.ac.ir; 3 Department of Horticulture, University of Mohaghegh Ardabili, Daneshgah Street, Ardabil 56199-11367, Iran; ghanbari66@uma.ac.ir; 4 Department of Plant Protection, University of Mohaghegh Ardabili, Daneshgah Street, Ardabil 56199-11367, Iran; mdavari@uma.ac.ir; 5 Food Packaging Lab, Department of Food, Environmental and Nutritional Sciences (DeFENS), University of Milan, Via Celoria 2, I-20133 Milan, Italy; daniele.carullo@unimi.it

**Keywords:** antifungal, coatings, emulsions, bacterial cellulose nanocrystals, essential oils

## Abstract

This work assessed the antimicrobial potential of natural essential oils (EOs) from cinnamon (CEO), *zataria* (ZEO), and *satureja* (SEO), applied natively or as coatings against *Penicillium expansum* and *Botrytis cinerea* during both in vitro and in vivo (on apple fruits) experiments. The induced inhibitory effect towards fungal growth, as a function of both EO type and concentration (75–1200 μL/L), was preliminarily investigated to select the most suitable EO for producing bacterial cellulose nanocrystals (BCNCs)/fish gelatin (GelA)-based emulsions. CEO and ZEO exhibited the best performances against *P. expansum* and *B. cinerea*, respectively. None of the pristine EOs completely inhibited the fungal growth and “disease severity”, properly quantified via size measurements of lesions formed on fruit surfaces. As compared to pristine CEO, coating emulsions with variable CEO concentration (75–2400 µL/L) curbed lesion spreading on apples, owing to the controlled CEO release during a 21-day temporal window. The strongest effect was displayed by BCNCs/GelA-CEO emulsions at the highest CEO concentration, upon which lesions on fruit skins were barely detectable. This work demonstrated the capability of EOs embedded in BCNCs/GelA-based nanocapsules to efficiently slow down microbial spoilage on postharvest fruits, thus offering viable opportunities for developing innovative antimicrobial packaging systems.

## 1. Introduction

Blue mold is a major postharvest disease of apples [1]. It is caused by *Penicillium expansum* and is limited almost entirely to stored fruit. It is of general occurrence, leading to approximately 80–90% of the total rot on stored apples, with loss accounting for up to 50% [2]. On the same line, grey mold is an infectious disease caused by *Botrytis cinerea*, a phytopathogenic fungus with a necrotrophic (e.g., that produces the death of the living tissue) lifestyle associated with over 200 crops worldwide, including stored fruit, which causes considerable losses [3]. While the utilization of chemical fungicides has been by far the most widely adopted strategy to control the above-mentioned infectious diseases, finding natural alternatives is of utmost importance to avoid the drawbacks associated with the long-term use of such substances [4].

Within this frame, many studies have successfully demonstrated that secondary metabolites or essential oils (EOs) of plants are effective for inhibiting fungal growth and are considered suitable natural alternatives to their chemical counterparts [5,6,7,8,9,10]. Specifically, in vitro and in vivo tests confirmed the effect of many essential oils on the inhibition or elimination of *P. expansum* and *B. cinerea* from apples and nectarines, respectively [11,12]. Among others, *Cinnamomum zeylanicum* [13,14,15,16], *Zataria multiflora* [17,18], and *Satureja khuzestanica* [19,20] were proven to be promising essential oils against fungal spoilage. However, EOs exhibit some main inherent shortcomings, such as the high volatility and sensitivity to oxygen and light during storage and processing operations [21]. Nanoencapsulation has been widely recognized as an effective technique to address the above issues, as well as to improve the solubility of EOs in water and mask their unfavorable flavor. In addition, nanoencapsulation represents a valid strategy to achieve a controlled release over time of the encapsulated active compounds via degradation and erosion of the outer polymer shell, while maintaining the physical stability of EOs [11,22,23,24,25]. 

The antifungal effect of some biopolymer-based coatings including plant EOs/extract against *P. expansum* and *B. cinerea* has been deeply investigated on different cultivars of apples. Among others, pullulan coating containing *Bergenia crassifolia* extract [26], chitosan coatings carrying olive oil residues [27], chitosan coatings and films loaded with different EOs [28], and starch–gellan coatings embedding thyme EO encapsulated in lecithin [29] were found to efficiently reduce the incidence and the severity of damaged spots on fruit surfaces, thus dramatically improving their shelf-life.

Gelatin has also been used to develop edible films and coatings due to its film-forming properties, biodegradability, biocompatibility, and good barrier properties to gases, oils, volatile compounds, and UV light [30,31]. At the same time, gelatin has been widely used as a surfactant due to its high stabilizing activity and good emulsifying properties [32]. Fish gelatin, in particular, is widely used as a replacement for mammalian gelatin to avoid the risks related to bovine spongiform encephalopathy (BSE) as well as to meet the requirements of Kosher and Halal dietary regulations [33]. 

To improve some functional properties of biopolymer films and coatings (e.g., barrier, mechanical, and stability properties) several nano-sized organic and inorganic particles can be used as the main biopolymer phase for the generation of biopolymer-based nanocomposites, which exhibit enhanced properties over their macro-sized counterpart (i.e., macrocomposites) [34]. Hence, synthesizing and applying active nanocomposite coatings including nano-sized entities such as nanofibers, nanoemulsions, and nanocapsules can represent a valid alternative to conventional approaches (e.g., chemical fungicides) to protect foods against pathogens and microbial spoilage during storage [35,36,37,38,39]. 

Cellulose nanocrystals (CNCs) extracted from both plants and bacteria, in particular, are highly promising for the generation of nanocomposite systems due to their exceptional features, such as high aspect ratio, high surface area, high crystallinity, and excellent mechanical properties [40]. 

Interestingly, in our recent study, we explored the effect of fish gelatin addition to bacterial cellulose nanocrystals (BCNCs) and cinnamon essential oil (CEO) on the stability, morphology, and encapsulation efficiency of nanoemulsions thereof, intended to potentially feed different food industry sectors [21]. Gelatin co-presence during nanoemulsion production led to a slight but significant improvement in the CEO encapsulation efficiency at pH = 5, although no further increases in system stability with respect to that yielded by BCNCs and CEO alone were recorded. Moreover, transmission electron microscopy (TEM) analyses, carried out in the same work, unveiled the remarkable surfactant activity of gelatin, which smoothly spread between the hydrophobic CEO nanodroplets and the polar BCNCs.

Therefore, after our previous study on fundamental aspects related to the gelatin-encapsulated CEO stabilized with BCNCs, in this work we decided to investigate the applicability of the antimicrobial emulsion coatings based on gelatin and BCNCs as a protective layer to inhibit the microbial growth on the surface of whole fruits, according to tests on inoculated apples. More specifically, three essential oils (e.g., thyme, cinnamon, and savory EOs) were used as natural antimicrobials applied either alone or embedded in BCNCs/fish gelatin nanocapsules against both *P. expansum* and *B. cinerea*. Specifically, the effect of both concentration and type of EO was examined by means of in vitro and in vivo tests, with the latter being carried out on apples belonging to the “*Red Delicious*” variety, properly chosen because of its large availability on the market. To the best of our knowledge, this is the first study on the effect of SEO, ZEO, and CEO essential oils against *P. expansum* and *B. cinerea* on *Red Delicious* apple by in vitro and in vivo experiments. 

## 2. Materials and Methods

### 2.1. Raw Materials and Chemicals

In this study, we used the following essential oils: *Cinnamomum zeylanicum*—CEO (Zardband Pharmaceuticals, Teheran, Iran), *Zataria multiflora Boiss*—ZEO (Tabib Daru Esfahan, Iran), and *Satureja khuzestanica*—SEO (Zardband Pharmaceuticals, Teheran, Iran). The chemical composition of each EO is displayed in the Supplementary Material (Table S2). Hand-harvested “*Red Delicious*” apples were obtained from a local orchard in Meshgin Shahr County (Ardabil Province, Iran), and selected according to a uniform size, maturity, color, and absence of injuries. The fungal strains used in this study were *Penicillium expansum* (IRAN 3009C) (Medicinal Plants and Drugs Research Institute of the Shahid Beheshti, University of Iran) and *Botrytis cinerea* (University of Tabriz). Type A (i.e., extracted by acid pretreatment) fish gelatin (GelA, Kosher, and Halal certified) with a gel strength of 200 Bloom was purchased from Weishardt (Graulhet, France), whereas bacterial cellulose was obtained from *Komagataeibacter sucrofermentans DSM 15973* (Leibniz Institute DSMZ-German Collection of Microorganisms and Cell Cultures, Braunschweig, Germany) under static conditions, as described in our previous work [41]. Sodium hypochlorite, potato dextrose agar (PDA) media, Tween 80, and sulfuric acid were purchased from Merck (Darmstadt, Germany).

All the adopted chemicals were of reagent grade and utilized without further purification.

### 2.2. Preparation of Pathogenic Fungal Isolates 

The fungal isolates were cultured on PDA in 90 mm Petri dishes and grown for 7 days (for *P. expansum*) and 10 days (for *B. cinerea*) at 25 ± 2 °C under light (12 h) and dark (12 h) incubation conditions. Isolate preparation was performed according to the method previously reported by Tahmasebi et al. [12]. For this purpose, the conidial suspension (0.1% *v*/*v* Tween 80 in 10 mL sterile-distilled water: 1000 µL/L) was first poured on culture plates. Afterward, conidia from fungal mycelium were released by scratching the surface of the PDA medium with a wire loop/scalpel. They were then transferred into microtubes and mixed with a vortex for 2–4 min to be separated from mycelium and subsequently suspended. The final solution was filtered through cheesecloth to remove extra fungal mycelium and finally obtain a pure conidia suspension. The conidia concentration was evaluated using a hemocytometer coupled with an optical microscope (Axioplan 2 Imaging, Carl Zeiss, Jena, Germany), according to the protocol reported in Table S3 of Supplementary Materials. The suspension was diluted with sterile water to the final concentration of approximately 1 × 10^6^ conidia/mL, and was then used for inoculating the fruits.

### 2.3. BCNCs/GelA-CEO Emulsion Preparation

**Figure 1:**
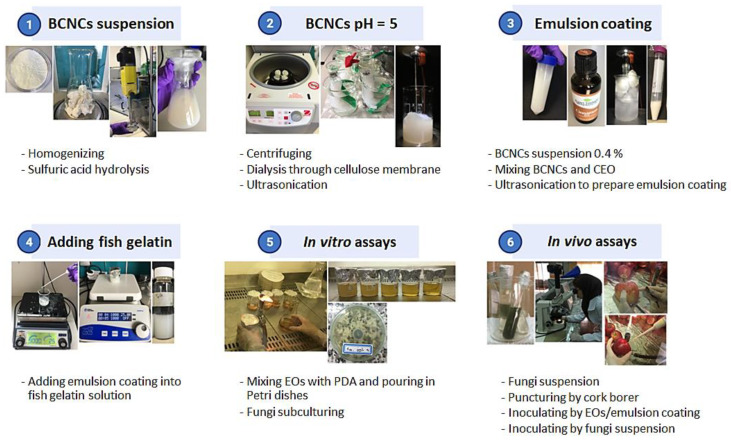
Flow chart of the three-step procedure of the BCNCs/GelA-CEO emulsions manufacturing.

The manufacturing of the BCNCs/GelA-CEO emulsions was a three-step procedure, according to the flow chart depicted in Figure 1. The first step involved the obtainment of the BCNCs from the macro-sized bacterial cellulose by acid hydrolysis using sulfuric acid, according to the method reported by Rovera et al. [41]. Briefly, 0.914 g of dry BC was added to 6.226 g of distilled water and 100 g of sulfuric acid (50% *w/w,* in distilled water). The solid particles were evenly dispersed using a DI 25 basic homogenizer with an S25 N–18 G dispersing tool (Ika-Werke GmbH & Co, Stanfen, Germany) at 9500 rpm for 3 min. The hydrolysis reaction was carried out by stirring at 55 °C for 2 h at 800 rpm. Afterward, the suspension was centrifuged for 50 min at 8000 rpm to facilitate the removal of excess sulfuric acid. After centrifugation, the supernatant was replaced with distilled water. After 5 washing cycles, the acid in excess was removed using dialysis tubes placed inside a beaker containing distilled water. The water was replaced every 4 h until the solution’s pH = 5. At this point, the BCNC water dispersions were put in a beaker and ultrasonicated for 5 min using a UP200St ultrasonicator (200 W, 26 kHz–Hielscher, Teltow, Germany) mounted with an S26d7D titanium sonotrode (surface area 42 mm^2^) at approximately 20 W (pulse: 25%, amplitude 30%) to achieve full nanocrystal dispersion. The samples were prepared at 0.4 wt.% BCNC dispersions (based on dry material), and stored at 4 °C.

In a second step, BCNCs were used to stabilize CEO emulsion according to a procedure reported in our previous work [21]. More specifically, 9 g of BCNC suspension (BCNC concentration 0.4% *w*/*w*) was added to various amounts of CEO (4.5, 9, 18, 36, 72, 108, 144, 216, 288 µL) and then ultrasonicated for 5 min at 40 W (pulse: 25%, amplitude 60%) in an ice bath to prevent sample overheating. 

Finally, gelatin was used as a surfactant at the interface between CEO nanodroplets and the surrounding medium [22]. A stock solution of fish gelatin (10% *w*/*w*) in water was prepared at 60 °C under constant stirring (800 rpm) for 2 h. Then, 8.25 g of distilled water were added to six vials, each containing 4.5 g of the stock solution. After decreasing the temperature to 40 °C, 2.25 g of each CEO emulsion was added dropwise into the six gelatin solutions and stirred for 15 min at 1000 rpm. The concentration of BCNCs and gelatin in the final BCNCs/GelA-CEO emulsion was 0.06% *w*/*w* and 3% *w*/*w* (0.6 mg/g and 30 mg/g), respectively. The final concentrations of CEO were 0.03, 0.06, 0.12, 0.24, 0.36, and 0.48% (*v*/*w*) (0.3, 0.6, 1.2, 2.4, 3.6 and 4.8 µL/g). The so obtained BCNCs/GelA-CEO emulsions were then used in both in vitro and in vivo experiments

### 2.4. In Vitro Antifungal Activity of EOs and BCNC/GelA-CEO Emulsions

Antifungal activity of both pristine EOs (CEO, ZEO, and SEO) and BCNC/GelA-CEO emulsions was investigated against *P. expansum* and *B. cinerea* in PDA, according to the flow chart reported in Figure S1a of Supplementary Material. For this purpose, 0.05% (500 µL/L) of Tween 80 was added to produce a 10% (10,000 µL/L) EO stock solution. After autoclaving at 1.5 atm and 121 °C, PDA and Erlenmeyer flasks were left under a laminar airflow hood to cool to around 40 °C. Then, 75, 150, 300, 600, and 1200 µL/L of EOs in PDA were prepared by adding the appropriate amounts of stock solution to the PDA flasks. For the BCNC/GelA-CEO emulsion, the same method was used. Then, the PDA media were poured onto 90 mm Petri dishes (each treatment was performed in three replicates) and left under a laminar airflow hood to cool and solidify. Then, the fungi discs were cut in 5 mm diameter using a cork borer from the 7-day-old cultures of *P. expansum* and 10-day-old cultures of *B. cinerea* isolates and placed in the middle of Petri dishes. For the control treatment, no EOs/emulsions were used in the PDA/Tween 80 mixture. Then, the Petri dishes were sealed using parafilm and incubated at 25 °C. After 24 h, the growth of cultures was monitored daily by measuring the diameter of the colonies until the control dishes were fully occupied by the fungi.

### 2.5. Antifungal Index

To quantify the inhibitive effect inferred by either EOs or BCNC/GelA-CEO emulsions towards fungal growth, as a function of the applied EO concentration, the antifungal index (AI) was calculated using the following equation [42]:

AI (%) = (C − T)/T · 100(1)

where C is the average diameter of the fungi colony of the control treatment, and T is the average diameter of the fungi colony of antifungal growth treatment. According to Equation (1), the AI parameter could vary between 0 (no inhibition) and 100% (maximum inhibition). The experiment was performed with 40 treatments and 3 replicates.

### 2.6. In vivo Antifungal Activity of EOs and BCNC/GelA-CEO Emulsions

According to the schematic sketch displayed in Figure S1b, fruits were firstly sanitized by immersion in a 2% sodium hypochlorite solution for 2 min, and then washed with sterile distilled water under a laminar flow hood and left for 1 h to dry. A sterile cork borer was used to make two opposite holes (approximately 4 mm in both diameter and depth) on the equatorial line of each apple. Then, 10 µL of EOs (75, 150, 300, 600, and 1200 µL/L) and emulsions (75, 150, 300, 600, 1200, 1800, and 2400 µL/L) were dropped into each hole. The extended concentration range for the emulsions was selected in consideration of the fact that a higher amount of EOs might be necessary against microbial spoilage because of the physical constraint represented by the BCNCs/GelA shell, which could affect the release of EOs. After inoculation, the fruits were left for 1 h to absorb the EO solution. The following step involved the addition of 10 µL of spore suspensions (1 × 10^6^ concentration) to each hole. For the positive control, a water–Tween 80 mixture was dropped into the holes without any further spore addition, whereas for the negative control a water–Tween 80 mixture was first poured into the holes and then inoculated with spore suspensions. Apples were placed in plastic sealed bags and incubated at 25 °C in the dark for 21 days until the surface of the negative controls was fully contaminated by the fungi. The antifungal efficiency of the proposed treatments was assessed by utilizing the approach followed by Zhang et al. [43]. Specifically, the diameters (mm) of the contaminated areas on treated fruits were measured and compared with those of negative controls, in correspondence to which they achieved their maximum size. Each treatment was performed 5 times.

### 2.7. Statistical Analysis

All collected results were treated in the form of a completely randomized design (CRD) and expressed as means ± standard deviations. Statistically significant differences among average values belonging to investigated samples were checked using a one-way analysis of variance (ANOVA) and the Tukey test. The significance level (*p*) was fixed at 0.05 and used for comparison of means using Minitab 18 statistical software (Coventry, UK).

## 3. Results

### 3.1. Antifungal Effects of EOs against P. expansum and B. cinerea (In Vitro Assay)

The effect of pristine CEO, ZEO, and SEO at different concentrations (75, 150, 300, 600, and 1200 µL/L) was investigated after 3, 9, and 15 days for *P. expansum* and 2, 5, and 7 days for *B. cinerea*, by monitoring the antifungal index (AI, %) (Table 1). Irrespective of the considered fungal species, all the EOs exhibited a straightforward antifungal effect against both *P. expansum* and *B. cinerea* for the highest EO concentrations (300–1200 μL/L for CEO and ZEO and 600–1200 μL/L for SEO) throughout the investigated period. This effect tended to gradually decrease, as demonstrated by the occurrence of significant (*p* < 0.05) decreases in the AI values as a function of time, especially when mild EO treatments were delivered. To this end, it must be inferred that both EO concentration and time had a significant effect up to 150 µL/L for CEO and ZEO, and up to 300 µL/L for SEO, which is linked to the release kinetics of EOs over time, as deeply discussed in our previous work [25].

Above these values, it was not possible to detect any difference among EOs in terms of AI values as a function of concentration and time, which can be plausibly attributed to an overloading effect of the essential oils (that is, the amount of EO loaded was more than necessary, insomuch as it totally inhibited fungi growth throughout the investigated period), thus causing full fungal disappearance from Petri dishes. However, further investigation is necessary to clarify this aspect. These results are consistent with the previous findings from Xing et al. [13], Behdani et al. [14], Fathi et al. [15], and Farzaneh et al. [19], which highlighted the linear relationship between applied EO concentration and the percentage of induced fungal growth inhibition.

Overall, as emerging from the statistical analysis in Table 1, CEO disclosed the best performance towards *P. expansum* growth, whereas *B. cinerea* biological activity seemed to be most efficiently curbed by the exploitation of ZEO. The difference between samples can be plausibly explained in terms of chemical composition of EOs. As reported in Table S2 of the Supplementary Material, cinnamaldehyde was the major component (~80%) of CEO, with minor components accounting for less than 5%. In the case of ZEO, thymol (32.68%), carvacrol (30.57%), p-cymene (8.94%), and γ-terpinene (5.76%) were the main components, with all the other components below 5%. Finally, carvacrol (38.43%), γ-terpinene (21.89%), p-cymene (16.55%), and α-terpinene (5.76%) were the major components of SEO. Hence, this different composition could have affected the different anti-fungal performance of the three EOs. The intimate reason for the different activity of these active molecules has been explained in terms of both chemical structure (e.g., presence and position of hydroxyl groups in phenolic compounds such as carvacrol and thymol, as well as the presence of carbonyl groups as in cinnamaldehyde) and possible synergisms between main and minor compounds in the same EO [44].

### 3.2. Antifungal Effects of EOs against P. expansum and B. cinerea (In Vivo Assay)

**Figure 2:**
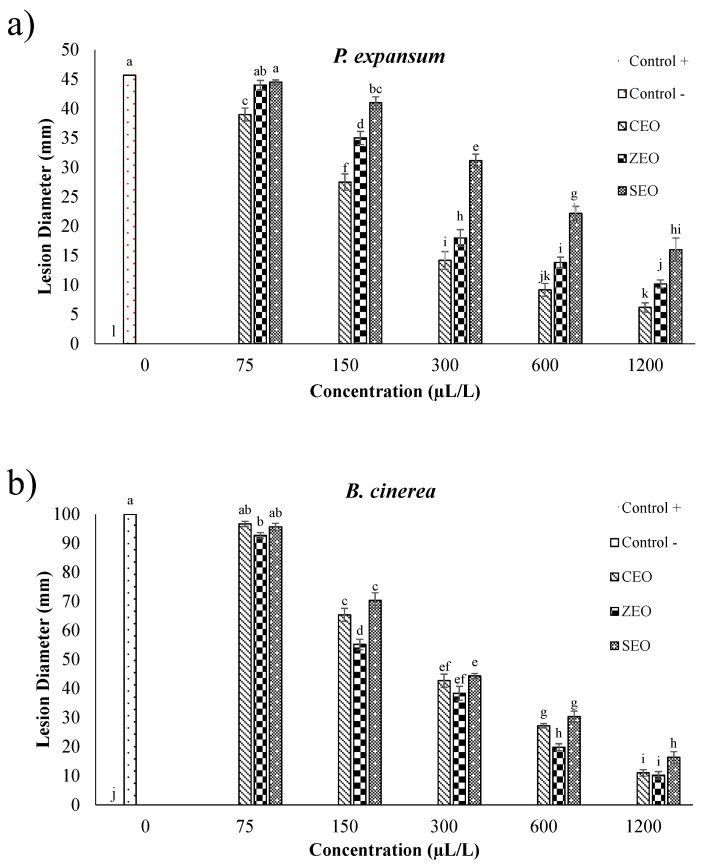
Effect of CEO, ZEO, and SEO treatments at different concentrations (75–1200 μL/L) against *P. expansum* (**a**) and *B. cinereal* (**b**), artificially inoculated into apple fruit (in vivo), after 21 days of storage at 25 °C. Each lesion diameter is an average of 15 replicates. Different letters above the bars denote a statistically significant difference according to the Tukey test (*p* < 0.05).

**Figure 3:**
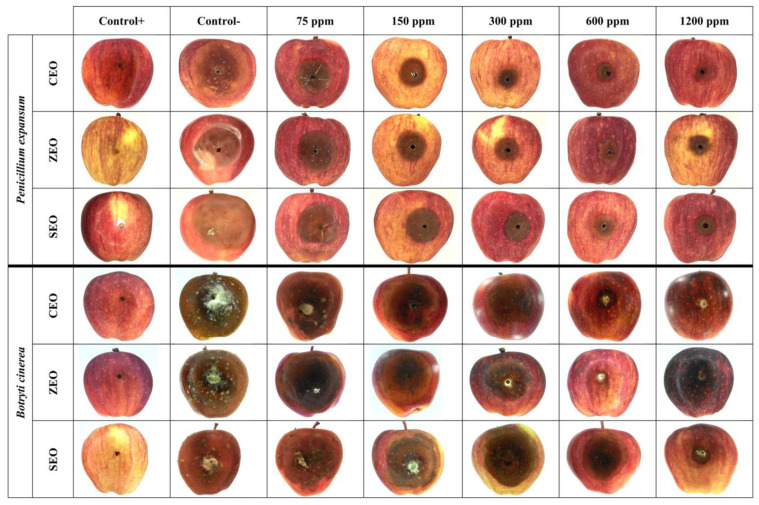
Digital images of apple surfaces treated with CEO, ZEO, and SEO at different concentrations (75–1200 μL/L) against *P. expansum* and *B. cinerea*, artificially inoculated into fruits, after 21 days of storage at 25 °C. Images of positive and negative controls are also reported.

According to the results reported in Figure 2 and as depicted in Figure 3, after 21 days of storage at 25 °C, an evident difference in the behavior of the applied EOs throughout the investigated range of concentrations was observed. In full agreement with data reported in Table 1 and in agreement with previous literature findings [20,45], a more prominent antifungal activity was in general observed by an increase in the EO concentration, regardless of the investigated fungal species. However, it is worth pinpointing that, for the same EO at a specific concentration, the detected antifungal activity was lower than that yielded during in vitro experiments, which is consistent with the results obtained by Etemadi et al. [17], Ranjbar et al. [46], and Taş and Karaca [47]. Among all the performed treatments, SEO had the lowest inhibitory effect, with lesion diameter values significantly (*p* < 0.05) higher than those shown upon CEO and ZEO application, for all the tested concentrations, and both fungi. Again, CEO and ZEO were found to display the highest inhibitory effect against *P. expansum* (Figure 2a) and *B. cinerea* (Figure 2b), respectively. In particular, for the CEO employed at 1200 µL/L, no evidence of fungal growth was observed for both fungi, though some evidence of lesion was still present on the fruit surface, as clearly seen in Figure 3. Overall, in the case of *P. expansum*, the lesion diameters did not exceed 45 mm in diameter in both control and treated apples (Figure 2a). Based on the obtained results, it can be stated that CEO acted better than SEO and, to a lesser extent, ZEO, especially in the case of *P. expansum*. Because *P. expansum* is the most deleterious fungus of apples during postharvest life, we decided to use CEO as the antifungal compound to be incorporated within the BCNC/GelA-based emulsion coatings during both in vitro and in vivo experiments against fungal spoilage (see next section).

Unfortunately, as illustrated in Figure 2 and Figure 3, no EOs could completely inhibit the fungi growth and occurrence of lesions on apples, even at the highest dose, as corroborated by the results obtained by Safari et al. [20], Fieira et al. [48], and Rupasinghe et al. [49]. These authors reported that this particular behavior is ascribable to both the properties of fruit’s tissue (e.g., amount of nutrients, pH level, natural phenolic compounds, etc.) and the components of EOs which, alone or synergistically with other components, can cause enhanced antifungal activity at a given concentration. Xing et al. [13] claimed that CEO at 0.1% concentration (1000 µL/L) had the least antifungal activity, whereas a 1% concentration (10,000 µL/L) had an excellent mycelial growth inhibition. Remarkably, greater concentrations, namely 2% (20,000 µL/L) and 3% (30,000 µL/L) concentrations, were able to completely inhibit the mycelial growth of *Rhizopus nigricans*, *Aspergillus flavus*, and *P. expansum* in jujube and orange fruits. Nevertheless, any emerging discrepancy arising from a mere comparison with our results could be attributed to the different applied EO concentrations, tested fruits, and amount of cinnamaldehyde in the essential oil. In a more recent study on the effect of SEO on controlling *P. expansum* spreading on apples after 20 days of storage [20], it was shown that the highest inhibitory potency was obtained with a concentration of 4000 µL/L, even though none of the considered doses can completely inhibit the fungus growth on apples. The differences between the results of the latter study and those collected in our work can be attributed to the fact that the effect of the essential oils depends on the intrinsic resistance of apple cultivars to *P. expansum* and *B. cinerea* [50].

### 3.3. Antifungal Effects of BCNCs/GelA-CEO Emulsions against P. expansum and B. cinerea (In Vitro and In Vivo Assays)

**Figure 4:**
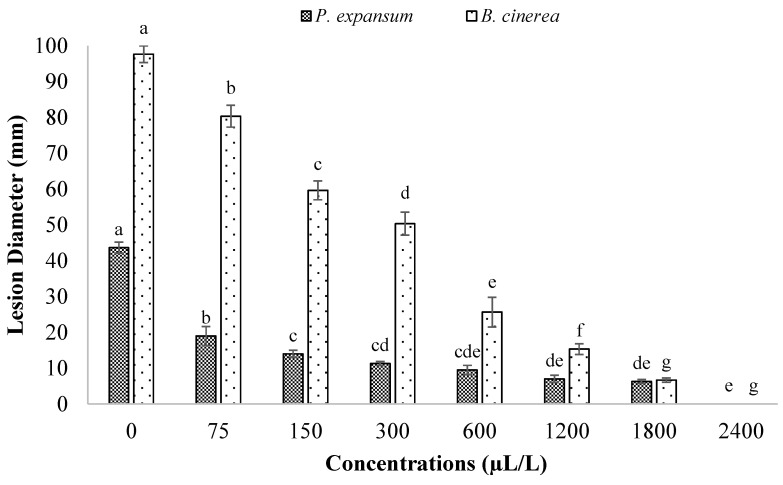
Effect of BCNCs/GelA−CEO treatments at different concentrations (75–2400 μL/L) against *P. expansum* and *B. cinerea*, artificially inoculated into apple fruit (in vivo), after 21 days of storage at 25 °C. Each lesion diameter is an average of 15 replicates. For any investigated fungus, different letters above the bars denote a statistically significant difference according to the Tukey test (*p* < 0.05).

**Figure 5:**
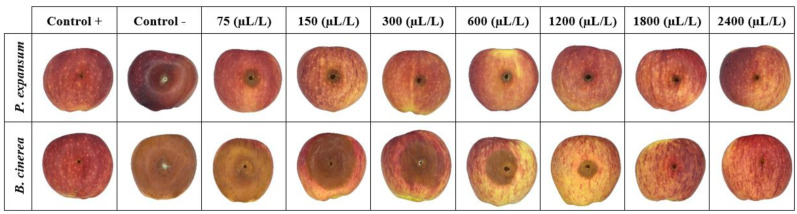
Digital images of apple surfaces treated with BCNCs/GelA−CEO emulsion coatings at different concentrations (75–2400 μL/L) against *P. expansum* and *B. cinerea*, artificially inoculated into fruits, after 21 days of storage at 25 °C.

The antifungal performance of the BCNCs/GelA-CEO emulsions is shown in Table 2 (in vitro assay) and Figure 4 and Figure 5 (in vivo assay). As far as the in vitro assay is concerned, CEO emulsions were shown to be effective in protecting against both fungi spreading. However, it should be noted that only CEO concentrations greater than 300 μL/L and 150 μL/L were capable of preventing *P. expansum* and *B. cinerea* growth, respectively, throughout the overall time window considered in this work, thus yielding a certain degree of stability from a microbiological point of view. As a matter of fact, at lighter doses, a significant (*p* < 0.05) reduction in AI values was recorded as long as the observation time was extended. In addition, by comparing the results of Table 2 with those obtained using the pristine CEO (Table 1), it is obvious that the emulsions did not allow the achievement of the same antifungal potential for *P. expansum*. For example, if we compare the AI values after two weeks at the lowest adopted concentration (75 µL/L), the pristine CEO yielded an AI of 29.29 ± 1.78, whereas the emulsion coating solutions gave an AI of 19.44 ± 4.4. Similarly, at a concentration of 150 µL/L, we observed an AI value of 55.37 ± 1.15 and 23.7 ± 3.5 for pristine and encapsulated CEO, respectively (Table S1 of the Supplementary Material). Reversely, a different scenario was opened up when *B. cinerea* was put under the spotlight. In particular, after one week, the encapsulated CEO worked decidedly better than the pristine CEO (AI = 44.63 ± 8.09 and 3.44 ± 0.5, respectively) at the lowest concentration (75 µL/L), whereas no statistical (*p* > 0.05) differences were detected at greater administered doses (Table S1).

With regards to the in vivo experiments, the preliminary trapping of CEO within a BCNCs/GelA matrix yielded a superior performance compared to pristine CEO. This finding was confirmed by comparing the lesion diameter values reported in Figure 2 and Figure 4, as well as from the apple surface photos displayed in Figure 3 and Figure 5. Specifically, the lesions on the apples’ surface were clearly observed throughout the experiments when using pristine CEO, especially in the case of *P. expansum* (Figure 3). Instead, in the case of CEO encapsulated in BCNCs/GelA, the size of the lesions abruptly decreased and, apparently, did not form on the apples’ surface at a CEO concentration exceeding 1200 µL/L or for both fungi. 

The different efficacy of CEO (pristine or encapsulated form) as revealed by in vivo experiments can be likely explained in terms of release kinetics of the active compound. Indeed, while pristine CEO was readily available for triggering an “instant” action, the encapsulated CEO was released in the surrounding medium according to a “controlled” mechanism, which allowed a continuous antifungal action over time to be achieved. Similar findings were collected in the study of Moreno et al. [51], who investigated the antifungal effect of gelatin-based edible coatings carrying ethanolic extract of propolis (PEE) against *P. expansum* and *B. cinerea* fungi inoculated into raspberries. The authors used two methods to embed PEE in the gelatin matrix: (i) PEE was directly added into the gelatin matrix, and (ii) PEE was nanoencapsulated using zein and then was added into the gelatin matrix. It was indisputably disclosed that the encapsulation improved the efficiency in reducing lesion symptoms on raspberry fruits.

In the big picture, when considering the overall performance of encapsulated CEO in terms of exerted antifungal activity, any effect possibly arising from other factors rather than the administered dose is worth being examined. For instance, fungi spores possess a negative charge that can interact via electrostatic forces with positively charged systems [52]. Because, in our work, gelatin at pH ~ 5 bears a positive charge, it can be eventually postulated that gelatin could have negatively affected the inhibition of fungi growth mediated by CEO. Nevertheless, such an adverse effect could have been partially compensated by the negatively charged BCNCs bearing sulfate groups. However, it should also be considered that the cellulose backbone can experience some degradation into short chains, cellobiose, and glucose by the extracellular enzymes of fungi (e.g., cellulase), which can absorb and process these products for their metabolism [41,53]. As demonstrated in a study on three species of *Penicillium*, the presence of cellulose can promote the growth of fungi, especially in the presence of water, which was also explained in terms of the higher affinity of fungi for polar and hydrophilic systems rather than apolar and hydrophobic ones [54].

According to the latter considerations, it clearly emerges that the determination of the suitable concentration of cellulosic materials outstands as a pivotal parameter to achieve improved antifungal properties in cellulose-based coatings [55].

### 3.4. Mechanism of Action of BCNCs/GelA-CEO Emulsion Coatings against P. expansum and B. cinerea 

The mode of EOs is linked to their ability to pass through the cell wall and penetrate the cell membrane across the lipid bilayer, thus causing an increase in the cell permeability, which finally leads to the cell death or inhibition of the sporulation and germination of fungi [56]. More specifically, it has been demonstrated that EOs interact with ergosterol, which is essential to maintain cell integrity, viability, function, and normal fungal growth [57]. Another mode of action involves the interaction of the phenolic compounds of EOs with the proteins in the cytoplasmatic membrane (porins) that can precipitate and lead to leakage of ions and other cell contents, causing the cell breakdown [58].

The effectiveness of CEO during in vivo experiments was lower than in the in vitro tests. This fact was also observed by Sapper et al. [29] and da Rocha Neto et al. [59] for apples. These authors explained this finding first considering that the interactions between EOs and fungal pathogens are modulated by the fruit host and the conditions in the wound, often resulting in reduced disease control ability. Second, the physicochemical aspects possibly influencing the release of EOs from the encapsulating coating should be taken into consideration. For example, the degree of coating plasticization, which depends on both the type and amount of plasticizer used in the coating formulation (e.g., glycerol, sorbitol, etc.) has a dramatic effect on the EOs actually available to exert an antimicrobial action. Finally, another important factor to consider is that (once laid on the fruit surface) EOs can somehow cause physiological perturbations in the fruit, decreasing the inherent defenses against fungi. In general, many factors could affect the biological activity of certain compounds when in contact with fruit tissue, and their biological activity depends on the complex host/antimicrobial compound/pathogen system [57]. Accordingly, it appears clear that the effectiveness of EOs cannot be anticipated by their antifungal activity in in vitro tests, as demonstrated in this work. 

## 4. Conclusions

Among the three different EOs tested in this study, CEO showed the best antifungal activity against *P. expansum*, being the most deleterious fungus of apples. BCNCs/GelA-CEO emulsions were demonstrated to work efficiently against both *P. expansum* and *B. cinerea* growth, with a better performance over the pristine (non-encapsulated) essential oil in the case of *P. expansum*. Moreover, the best fungicide effect of BCNCs/GelA-CEO was obtained at a CEO concentration of 2400 µL/L.

Since hydrophilic coating matrices are well suited for fruits, owing to their solubility in water and ease of removal by simple washing, additional studies focused on fulfilling the right balance between emulsions’ performance (e.g., stability, wettability, controlled release of the active compound, etc.) and antifungal effect are strictly required to individuate the optimal formulation for the shelf-life extension goal, as well as to preserve fruit freshness.

## Figures and Tables

**Table 1:** Antifungal index (AI,%) for CEO, ZEO, and SEO treatments against *P. expansum* and *B. cinerea* (in vitro assay). For any investigated fungus, different right-side lowercase and uppercase letters within the same column denote significant differences (*p* < 0.05) among AI values due to the effect of employed EO concentration and EO type, respectively. When reported, different left-side lowercase letters within the same row indicate significant differences (*p* < 0.05) among AI values only due to the effect of observation time.

		*P. expansum*			*B. cinerea*		
	Concentration [(μL/L)	Time (Days)			Time (Days)		
		3	9	15	2	5	7
CEO	75	^b^ 44.0 ± 2.6 ^a,C^	^b^ 38.7 ± 2.5 ^a,B^	^a^ 29.3 ± 1.8 ^a,C^	^b^ 18.1 ± 3.5 ^a,AB^	^a^ 5.4 ± 1.2 ^a,A^	^a^ 3.4 ± 0.5 ^a,A^
	150	^b^ 67.2 ± 4.6 ^b,C^	^b^ 64.7 ± 5.3 ^b,C^	^a^ 55.4 ± 1.1 ^b,C^	^b^ 100.00 ± 0.0 ^b,B^	^a^ 90.4 ± 0.3 ^b,B^	^a^ 89.6 ± 1.3 ^b,B^
	300	100.0 ± 0.0 ^c,B^	100.0 ± 0.0 ^c,B^	100.0 ± 0.0 ^c,B^	100.0 ± 0.0 ^b,B^	100.0 ± 0.0 ^c,B^	100.0 ± 0.0 ^c,B^
	600	100.0 ± 0.0 ^c,A^	100.0 ± 0.0 ^c,A^	100.0 ± 0.0 ^c,A^	100.0 ± 0.0 ^b,A^	100.0 ± 0.0 ^c,A^	100.0 ± 0.0 ^c,A^
	1200	100.0 ± 0.0 ^c,A^	100.0 ± 0.0 ^c,A^	100.0 ± 0.0 ^c,A^	100.0 ± 0.0 ^b,A^	100.0 ± 0.0 ^c,A^	100.0 ± 0.0 ^c,A^
ZEO	75	^b^ 22.1 ± 1.8 ^a,B^	^ab^ 17.2 ± 2.5 ^a,A^	^a^ 15.2 ± 1.7 ^a,B^	11.9 ± 6.4 ^a,A^	14.6 ± 2.1 ^a,B^	11.8 ± 1.7 ^a,C^
	150	^c^ 46.2 ± 1.2 ^b,B^	^b^ 40.1 ± 1.0 ^b,B^	^a^ 34.1 ± 1.3 ^b,B^	100.0 ± 0.0 ^b,B^	100.0 ± 0.0 ^b,C^	100.0 ± 0.0 ^b,C^
	300	100.0 ± 0.0 ^c,B^	100.0 ± 0.0 ^c,B^	100.0 ± 0.0 ^c,B^	100.0 ± 0.0 ^b,B^	100.0 ± 0.0 ^b,B^	100.0 ± 0.0 ^b,B^
	600	100.0 ± 0.0 ^c,A^	100.0 ± 0.0 ^c,A^	100.0 ± 0.0 ^c,A^	100.0 ± 0.0 ^b,A^	100.0 ± 0.0 ^b,A^	100.0 ± 0.0 ^b,A^
	1200	100.0 ± 0.0 ^c,A^	100.0 ± 0.0 ^c,A^	100.0 ± 0.0 ^c,A^	100.0 ± 0.0 ^b,A^	100.0 ± 0.0 ^b,A^	100.0 ± 0.0 ^b,A^
SEO	75	^b^ 13.8 ± 1.2 ^a,A^	^ab^ 12.1 ± 2.5 ^a,A^	^a^ 8.5 ± 1.7 ^a,A^	^c^ 25.4 ± 1.7 ^b,B^	^b^ 19.2 ± 1.0 ^a,C^	^a^ 7.4 ± 0.4 ^a,B^
	150	^b^ 33.8 ± 1.2 ^b,A^	^b^ 31.5 ± 1.7 ^b,A^	^a^ 25.5 ± 2.2 ^b,A^	^a^ 52.5 ± 6.1 ^c,A^	^b^ 65.4 ± 4.2 ^b,A^	^a^ 48.5 ± 3.9 ^b,A^
	300	78.0 ± 4.3 ^c,A^	74.6 ± 4.0 ^c,A^	69.2 ± 3.9 ^c,A^	^a^ 7.4 ± 0.4 ^a,A^	^c^ 83.8 ± 1.0 ^c,A^	^b^ 81.3 ± 1.1 ^c,A^
	600	100.0 ± 0.0 ^d,A^	100.0 ± 0.0 ^d,A^	100.0 ± 0.0 ^d,A^	100.0 ± 0.0 ^d,A^	100.0 ± 0.0 ^d,A^	100.0 ± 0.0 ^d,A^
	1200	100.0 ± 0.0 ^d,A^	100.0 ± 0.0 ^d,A^	100.0 ± 0.0 ^d,A^	100.0 ± 0.0 ^d,A^	100.0 ± 0.0 ^d,A^	100.0 ± 0.0 ^d,A^

**Table 2:** Antifungal index (AI,%) of BCNCs/GelA emulsion treatments (CEO concentration = 75–1200 μL/L) against *P. expansum* and *B. cinerea* (in vitro assay). For any investigated fungus, different lowercase letters within the same column denote significant differences (*p* < 0.05) among AI values due to the effect of employed CEO concentration, whereas different uppercase letters within the same row express significant differences (*p* < 0.05) amongst AI values due to the effect of observation time. Legend: - = not measured.

	Concentration (μL/L)	Time (Days)						
		2	5	7	9	13	15	20
*P. expansum*	75	82.4 ± 2.0 ^a,E^	74.1 ± 17.1 ^a,DE^	56.7 ± 1.5 ^a,CD^	44.8 ± 1.5 ^a,BC^	28.9 ± 5.4 ^a,AB^	19.4 ± 3.9 ^a,A^	12.9 ± 0.5 ^a,A^
	150	95.9 ± 6.3 ^b,E^	76.5 ± 6.7 ^ab,D^	67.8 ± 6.9 ^a,CD^	54.6 ± 7.6 ^b,C^	39.2 ± 2.0 ^a,B^	23.7 ± 3.2 ^a,A^	20.0 ± 1.0 ^ab,A^
	300	100.0 ± 0.0 ^c,D^	94.4 ± 8.6 ^ab,D^	92.6 ± 11.5 ^b,D^	87.4 ± 9.9 ^c,CD^	62.9 ± 9.6 ^b,BC^	45.8 ± 9.0 ^b,AB^	31.1 ± 10.6 ^b,A^
	600	100.0 ± 0.0 ^c,A^	100.0 ± 0.0 ^b,A^	100.0 ± 0.0 ^b,A^	100.0 ± 0.0 ^c,A^	100.0 ± 0.0 ^c,A^	100.0 ± 0.0 ^c,A^	100.0 ± 0.0 ^c,A^
	1200	100.0 ± 0.0 ^c,A^	100.0 ± 0.0 ^b,A^	100.0 ± 0.0 ^b,A^	100.0 ± 0.0 ^c,A^	100.0 ± 0.0 ^c,A^	100.0 ± 0.0 ^c,A^	100.0 ± 0.0 ^c,A^
*B. cinerea*	75	69.3 ± 3.0 ^a,D^	54.3 ± 4.6 ^a,CD^	44.6 ± 7.2 ^a,BC^	31.7 ± 6.1 ^a,B^	5.2 ± 0.5 ^a,A^	-	-
	150	95.4 ± 7.2 ^b,B^	92.8 ± 11.2 ^b,B^	90.7 ± 14.4 ^b,B^	72.6 ± 16.1 ^b,B^	12.6 ± 3.2 ^b,A^	-	-
	300	100.0 ± 0.0 ^b,A^	100.0 ± 0.0 ^b,A^	100.0 ± 0.0 ^c,A^	100.0 ± 0.0 ^c,A^	100.0 ± 0.0 ^c,A^	-	-
	600	100.0 ± 0.0 ^b,A^	100.0 ± 0.0 ^b,A^	100.0 ± 0.0 ^c,A^	100.0 ± 0.0 ^c,A^	100.0 ± 0.0 ^c,A^	-	-
	1200	100.0 ± 0.0 ^b,A^	100.0 ± 0.0 ^b,A^	100.0 ± 0.0 ^c,A^	100.0 ± 0.0 ^c,A^	100.0 ± 0.0 ^c,A^	-	-

## Data Availability

Data are contained within the article and the Supplementary Material.

## Supplementary Materials

The following supporting information can be downloaded at: https://www.mdpi.com/article/10.3390/foods11111602/s1, Table S1: In vitro and in vivo antifungal results of pristine CEO compared to BCNCs/GelA-CEO emulsions against *P. expansum* and *B. cinerea*. For any investigated test/fungus pair, different lowercase letters within the same row denote significant differences (*p* < 0.05) between mean values due to the different modes of administration of CEO (pristine or encapsulated). Table S2. Chemical composition of CEO, ZEO, and SEO by GC-MS analysis (adapted from Tahmasebi et al., 2020 [12]). Table S3. Schematic description of the protocol used for cell counting (adapted from Selvakumaran & Jell, 2005 [60]). Figure S1. Schematic representation of the procedure used to assess the *in vitro* (a) and *in vivo* (b) antifungal activity of both pristine EOs (CEO, ZEO, and SEO) and BCNC/GelA-CEO emulsions.
