# Supplementary material for: Production of Innovative Essential Oil-Based Emulsion Coatings for Fungal Growth Control on Postharvest Fruits
Source: Foods. 2022 May 29;11(11):1602. doi: 10.3390/foods11111602 (PMC9180006; doi:10.3390/foods11111602)
Supplement: Supplementary file 1 [file foods-11-01602-s001.zip › foods-1719097-supplementary.pdf]

Table S1. *In vitro* and *in vivo* antifungal results of pristine CEO compared to BCNC/GelA-CEO emulsions against *P. expansum* and *B. cinerea*. For any investigated test/fungus pair, different lowercase letters within the same row denote significant differences ($p < 0.05$) between mean values due to the different modes of administration of CEO (pristine or encapsulated).

	Concentrations [μL/L]	In vitro test (AI after 2 weeks, %)		In vivo test (Lesion diameter after 21 days, mm)	
		Pristine CEO	BCNC/GelA-CEO emulsions	Pristine CEO	BCNC/GelA-CEO emulsions
P. expansum	75	29.3 ± 1.8[b]	19.4 ± 4.4[a]	39.1 ± 1.0[b]	19.0 ± 2.6[a]
	150	55.4 ± 1.1[b]	23.7 ± 3.5[a]	27.5 ± 2.1[b]	14.0 ± 1.0[a]
	300	100.0 ± 0.0[b]	45.7 ± 10.1[a]	14.2 ± 0.5[b]	9.5 ± 0.5[a]
	600	100.0 ± 0.0[a]	100.0 ± 0.0[a]	9.2 ± 1.3[a]	7.0 ± 1.3[a]
	1200	100.0 ± 0.0[a]	100.0 ± 0.0[a]	6.2 ± 2.2[a]	6.3 ± 1.0[a]
		In vitro test (AI after 2 weeks, %)		In vivo test (Lesion diameter after 21 days, mm)	
B. cinerea	75	3.4 ± 0.5[a]	44.6 ± 8.1[b]	96.7 ± 3.2[b]	80.3 ± 3.1[a]
	150	89.6 ± 1.3[a]	90.7 ± 16.0[a]	65.3 ± 2.4[b]	59.6 ± 2.6[a]
	300	100.0 ± 0.0[a]	100.0 ± 0.0[a]	42.7 ± 6.4[a]	50.3 ± 3.2[a]
	600	100.0 ± 0.0[a]	100.0 ± 0.0[a]	27.2 ± 4.3[a]	25.3 ± 4.1[a]
	1200	100.0 ± 0.0[a]	100.0 ± 0.0[a]	11.9 ± 5.1[a]	15.3 ± 1.5[a]

Table S2. Chemical composition of CEO, ZEO, and SEO by GC-MS analysis (adapted from Tahmasebi et al., 2020).

components	CEO (%)	ZEO (%)	SEO (%)	Retention index
α-Thujene	1.53	3.63	2.32	933
α-pinene	-	0.43	2.64	938
Benzaldehyde	0.37	-	-	944
Camphene	-	0.15	-	946
3-Octanone	-	0.14	-	966
β-pinene	-	0.39	1.83	973
β-Myrcene	-	0.71	2.47	981
α-Phellandrene	-	0.13	-	990
3-Carene	0.65	-	-	998
α-Terpinene	-	0.52	**5.76**	1008
p-Cymene	1.54	**8.94**	**16.55**	1009
Limonene	-	0.56	1.45	1018
β-Phellandrene	0.37	-	-	1024

Eucalyptol	1.15	0.43	-	1034
γ-Terpinene	0.08	**5.96**	**21.89**	1048
Linalool	-	1.13	-	1098
β-Terpineol, cis-	-	0.1	-	1135
α-Terpineol	0.08	1.54	-	1189
Thymol methyl ether	-	0.67	-	1216
Carvacrol methyl ether	-	0.98	-	1225
Carvone	-		1.21	1243
Linalool acetate	2.65	-	-	1248
Cinnamaldehyde	**80.82**	-	-	1263
Thymol	-	**32.68**	1.94	1281
Carvacrol	-	**30.57**	**38.43**	1294

components	CEO (%)	ZEO (%)	SEO (%)	Retention index
Thymol acetate	-	1.45	-	1332
Carvacrol acetate	-	1.78	-	1348
Caryophyllene, (Z)-	-	2.38	-	1404
Aromadendrene	-	1.21	-	1441
Caryophyllene	3.57	-	-	1456
(Z)-Cinnamic acid	0.12	-	-	1471
γ-Bisabolene, (E)-	-	0.8	2.32	1523
Spathulenol	-	0.83	-	1563
2H-1- Benzopyran-2-one	0.1	-	-	1581
Cinnamyl acetate	0.14	-	-	1635
Caryophyllene oxide	0.03	0.78	-	1961
**Total**	97.66	98.46	98.81	

Table S3. Schematic description of the protocol used for cell counting (adapted from Selvakumaran & Jell, 2005).

1. Make a cell suspension according to the sub-culture protocol.

2. Prepare a hemocytometer and coverslip by spraying with 70% alcohol and drying with a clean tissue.

3. Fix a coverslip on the hemocytometer by moistening the coverslip with water or exhaled air, slide the coverslip over the chamber, and move back and forth exerting slight pressure until Newton's rings (rainbow-colored rings) appear.

4. Place 0.2 ml of a suitable cell suspension (in complete medium) in a sealed container.

5. Add 0.2 ml of 0.4% trypan blue stain and mix thoroughly (dilution factor of 2).

6. Allow to stand for 2–3 minutes at 15 to 30 °C (room temperature). Prolonged exposure to trypan blue kills the cells.

7. With a pipette, fill both chambers of the hemocytometer. Do not over- or under-fill the chambers. Make sure that there are no air bubbles.

8. Under a microscope count the number of viable (unstained) and non-viable (stained blue) cells in eight-ten 4 × 4 squares or 0.1 cm^2 area. Count the cells in the squares and touch the left and top middle line.

9. Calculate the number of viable and non-viable cells/ml using the formula given below.

Number of viable cells/ml =	The average number of viable cells per 0.1 cm^2 area $\times 10^4$ (correction factor for volume of shaded area) $\times$ 2 (dilution factor, 0.2 ml of cell suspension in 0.4 ml)

10. Then, by having the total volume of fungi suspension and multiplying by the number of cells in 1 ml obtained from the above formula, the final concentrations will be obtained.

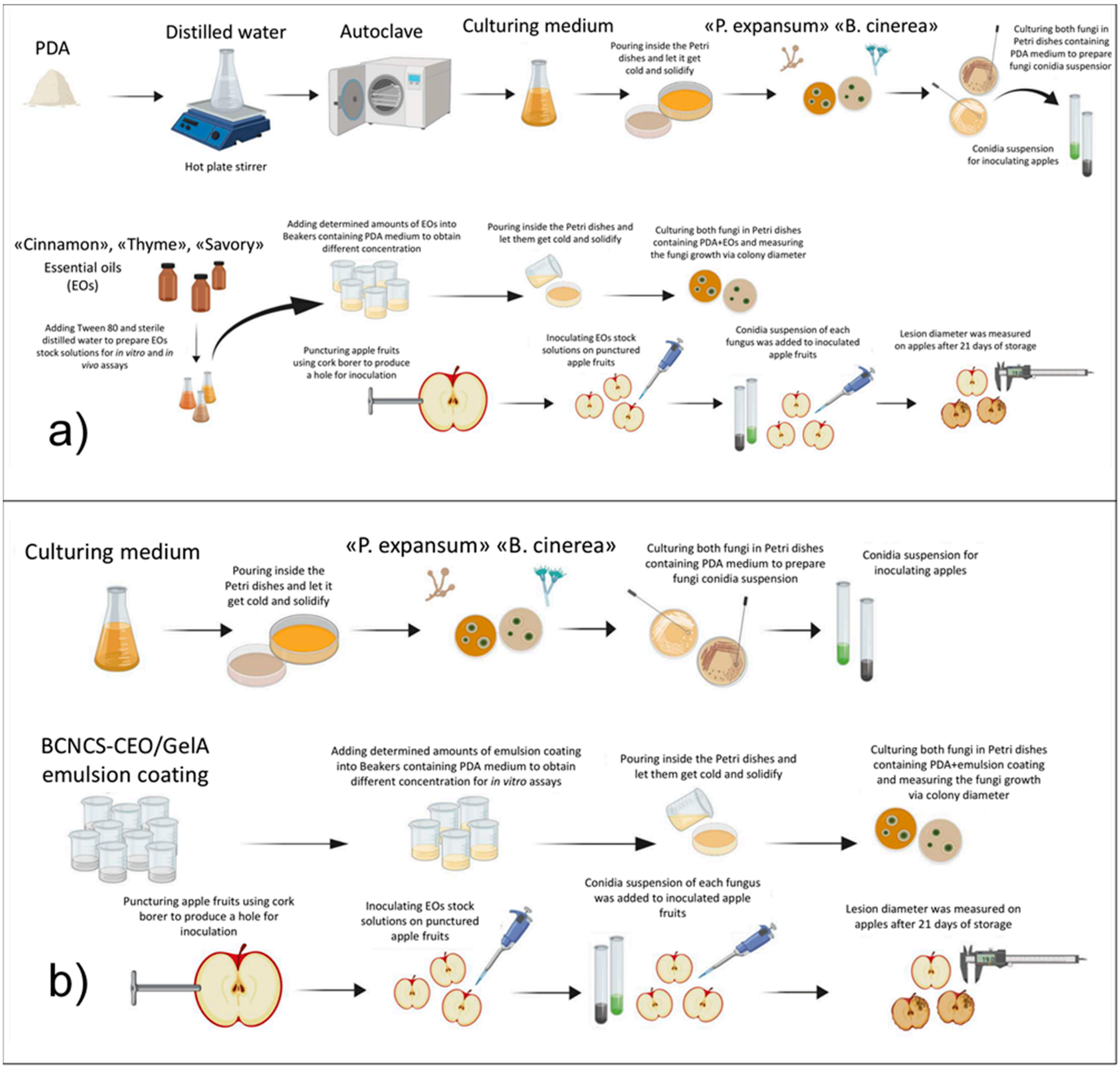

Figure S1. Schematic representation of the procedure used to assess the *in vitro* (a) and *in vivo* (b) antifungal activity of both pristine EOs (CEO, ZEO, and SEO) and BCNC/GelA-CEO emulsions.

References

1. Tahmasebi, M.; Golmohammadi, A.; Nematollahzadeh, A.; Davari, M.; Chamani, E. Control of nectarine fruits postharvest fungal rots caused by *Botrytis cinerea* and *Rhizopus stolonifer* via some essential oils. *J. Food Sci. Technol.* **2020**, *57*, 1647–1655. https://doi.org/10.1007/s13197-019-04197-4
2. Selvakumaran, J.; Jell, G. A guide to basic cell culture and applications in biomaterials and tissue engineering. In Biomaterials, Artificial Organs and Tissue Engineering, 1st ed.; Hench, L.L., Jones, J.R., Eds.; Woodhead Publishing Series in Biomaterials, 2005; pp. 215–226. https://doi.org/10.1533/9781845690861.4.215
